# Injectable Gamboge-Based In Situ Gel for Sustained Delivery of Imatinib Mesylate

**DOI:** 10.3390/gels9090737

**Published:** 2023-09-12

**Authors:** Kritamorn Jitrangsri, Ei Mon Khaing, Torsak Intaraphairot, Thawatchai Phaechamud, Jongjan Mahadlek

**Affiliations:** 1 Department of Chemical Engineering and Pharmaceutical Chemistry, School of Engineering and Technology, Walailak University, Nakhon Srithammarat 80160, Thailand; kritamorn.ji@mail.wu.ac.th; 2 Natural Bioactive and Material for Health Promotion and Drug Delivery System Group (NBM Group), Faculty of Pharmacy, Silpakorn University, Nakhon Pathom 73000, Thailand; eimonkhaing10@gmail.com (E.M.K.); intaraphairot_t@su.ac.th (T.I.); phaechamud_t@su.ac.th (T.P.); 3 Program of Pharmaceutical Engineering, Faculty of Pharmacy, Silpakorn University, Nakhon Pathom 73000, Thailand; 4 Department of Biopharmacy, Faculty of Pharmacy, Silpakorn University, Nakhon Pathom 73000, Thailand; 5 Department of Industrial Pharmacy, Faculty of Pharmacy, Silpakorn University, Nakhon Pathom 73000, Thailand; 6 Pharmaceutical Intellectual Center “Prachote Plengwittaya”, Faculty of Pharmacy, Silpakorn University, Nakhon Pathom 73000, Thailand

**Keywords:** gamboge, gambogic acid, imatinib mesylate, in situ gel, cytotoxicity

## Abstract

The aim of this study was to prepare and characterize the imatinib mesylate (IM)-loaded gamboge-based ISG system for local administration of an anticancer agent against colorectal carcinoma. The ISG formulations were prepared in dimethyl sulfoxide (DMSO) and *N*-methyl-2-pyrrolidone (NMP). The physicochemical properties, drug release profile, and cytotoxicity of the developed formulations were assessed. The developed ISG demonstrated Newtonian flow behavior with acceptable rheological and mechanical properties. The viscosity of the developed ISG, measured at less than 80 cP, and the applied forces of less than 50 N·mm, indicated easy administration using clinical injection techniques. Upon contact with an aqueous phase, the ISG immediately formed a porous cross-sectional structure, enabling sustained release of IM over 14 days. The release profile of IM was fitted to the quasi-Fickian diffusion mechanism, and the release rate could be controlled by the types of solvent and the amount of IM content. The developed IM-loaded gamboge ISG effectively inhibited colorectal cancer cells, including HCT116 and HT29 cell lines, with less than 20% cell viability observed at a concentration of 1% *w*/*w* IM after 2 days of incubation. This suggests that the developed ISG may potentially serve as an injectable system for localized anticancer delivery against colorectal cells, potentially reducing the side effects of systemic chemotherapy and improving patient adherence.

## 1. Introduction

**Figure 1:**
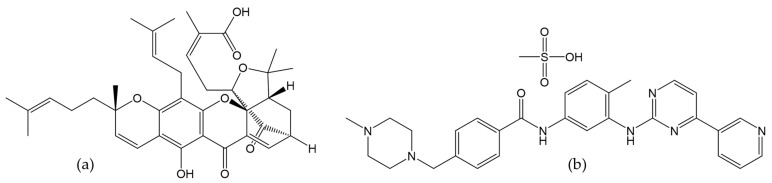
Chemical structure of gambogic acid (GA) (**a**) and imatinib mesylate (IM) (**b**).

Gamboge is a resin extracted from the *Garcinia xanthochymus* tree, a member of the Clusiaceae (Guttiferae) family and the Garcinia genus [1]. This plant is native to northern Thailand and Myanmar [2]. Gamboge resin contains caged polyprenylated xanthones, which exhibit various pharmacological activities, including anti-cancer, anti-HIV, anti-bacterial, anti-inflammatory, and neurotrophic effects [3,4]. Gambogic acid (GA) (Figure 1a) is a major component of the gamboge resin and possesses a 4-oxa-tricyclo [4.3.1.0^3,7^]dec-2-one scaffold that shows promising antitumor, antiangiogenic, and antimetastatic properties against multiple cancer cell lines [5,6]. The antitumor mechanism of GA has been found to involve apoptosis induction through increased expression of executor caspase-8 [7]. GA underwent a phase-II clinical trial in China, which indicated a favorable safety profile at the administered dose [8]. However, its limited water solubility, short half-life, poor stability, and inflammatory response have hindered its clinical superiority evaluation [9].

Imatinib mesylate (IM) or a-(4-Methyl-1-piperazinyl)-30-{[4-(3-pyridyl)-2-pyrimidinyl]amino}-*p*-tolu-*p*-toluidide methanesulfonate is a chemical compound with the empirical formula C_29_H_31_N_7_O·CH_4_O_3_S (Figure 1b) [10]. IM is poorly soluble in neutral or alkaline pH solutions but very soluble in water at a pH below 5.5, and it is freely soluble in dimethyl sulfoxide (DMSO) and slightly soluble in methanol and ethanol [11]. This compound selectively inhibits protein tyrosine kinase activity and has been shown to be effective in treating chronic myeloid leukemia and gastrointestinal stromal tumors [12,13]. Common adverse reactions to IM include muscle cramping and myalgias [14]. Rare but serious complications include intracranial bleeding, heart failure, and left ventricular dysfunction [14,15].

Today, various types of novel drug delivery systems have been established [16]. In situ forming gel (ISG) is a drug delivery system that can transition from a liquid-like dosage form to a semisolid state upon administration at the target site [17,18]. This type of drug delivery system can be categorized into three types: phase-separation systems (induced by changes in temperature, solvent, and pH), cross-linkage systems (achieved through photo, chemical, and physical processes), and solidifying organo-gels (induced by changes in solubility) [19]. To prepare ISGs, the drug is dissolved in a bioresorbable polymeric solution in a biocompatible vehicle. Upon addition to an aqueous solution, the solvent is removed from the polymer, resulting in a semisolid matrix that entraps the drug compound. Over time, the many drugs are released through diffusion and progressive polymer degradation [20,21,22]. An ideal in situ gel former should possess low water solubility but can dissolve in water-miscible organic solvents [23]. Therefore, gamboge resin is a promising candidate for ISG formation due to these desirable properties. The incorporation of an active compound to study the controlled release property from gamboge resin-based ISG has not been established. In addition, given the selective target-based cancer therapy with a low incidence of side effects of IM [24], along with the desirable physicochemical properties, IM was selected as a model drug for the gamboge resin-based ISG study.

The aim of this study was to develop a novel ISG drug delivery platform using gamboge resin as a matrix former with IM-loading for local anticancer delivery against colorectal carcinoma, and investigate the relevant physicochemical characterization, drug release, and cytotoxicity of the developed formulation. The success of this work holds promise as an injectable system for targeted anticancer delivery against colorectal cells.

## 2. Results and Discussion

### 2.1. Gamboge-Based ISGs

#### 2.1.1. Gel Formation

**Figure 2:**
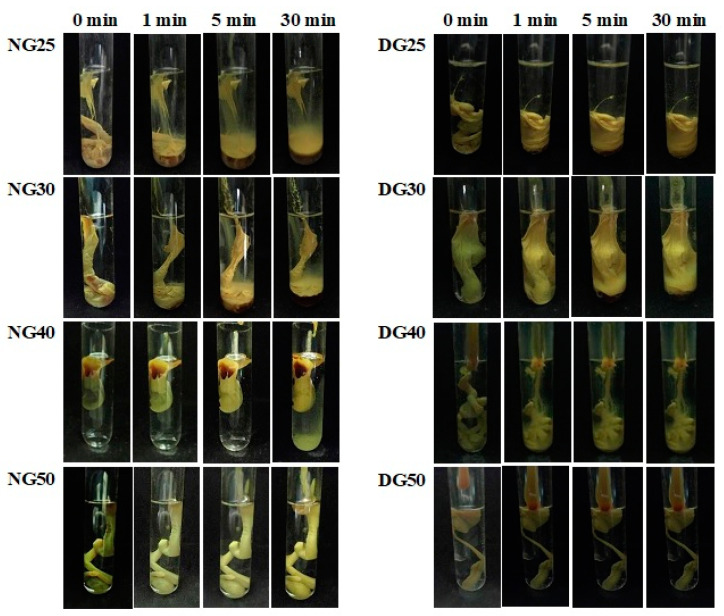
Gel formation of 25–50% *w*/*w* gamboge-based ISGs in NMP (**left**) and DMSO (**right**).

The gelation behavior of gamboge-based ISGs was investigated using different concentrations of gamboge (25–50% *w*/*w*) in PBS (pH 7.4), as illustrated in Figure 2. Upon contact with PBS, a solid-like matrix was immediately formed, indicating phase-inversion or phase-separation from the movement outward of DMSO and NMP from gamboge into the aqueous phase due to their miscibility and high affinity [25]. Notably, higher concentrations of gamboge resulted in a faster matrix formation, corresponding with previous reports [26].

Interestingly, at 25% *w*/*w* concentrations of gamboge-based ISGs, the matrix formation was not complete within 30 min when using NMP as the solvent. This delay in matrix formation can be attributed to the lower polarity of NMP (Log P −0.38) compared to DMSO (Log P −1.3), which slows down the gelation process [27]. It is well known that the higher hydrophobicity of the system leads to a reduced exchange rate of solvent, resulting in the formation of a sponge-like structure in ISGs [28].

This observation is consistent with previous studies that reported a faster ISG formation from higher polarity solvents, such as DMSO, compared to 2-pyrrolidone for bleached shellac scaffolds [29]. Similarly, rosin-based ISGs also formed more rapidly and produced thicker gels over time when DMSO was used as the solvent compared to NMP [30].

#### 2.1.2. pH, Viscosity, and Injectability

Table 1 shows that the pH of gamboge-based in situ gels (ISGs) in NMP was slightly higher than in DMSO. The higher gamboge content in the formulation reduced the pH values due to the increase in carboxylic acid, which is the functional group of the major compound in gamboge resin, gambogic acid [31].

The viscosity of gamboge-based ISGs in NMP was also slightly higher than that of DMSO, especially at a lower gamboge content (25–30% *w*/*w*). However, it showed significantly larger viscosity values in DMSO at a higher gamboge content (40–50% *w*/*w*) (*p* < 0.05) (Table 1). This is caused by the higher affinity of gamboge for DMSO, which leads to the formation of intermolecular bonds between solvent and solute molecules, resulting in higher viscosity values for the formulation [32]. The viscosity of gamboge-based ISGs in NMP was significantly lower compared to the viscosity of rosin/cinnamon oil-based ISGs in our previous study [30], indicating the easier injection of gamboge-based ISGs.

The injectability of the formulations refers to the force needed to expel the solution from the syringe, a parameter typically dependent on the viscosity values. Therefore, the injectability force of ISGs in DMSO was higher than that of NMP (Table 1). However, the highest injectability force among the gamboge-based ISGs was 1.38 ± 0.07 N, which is still less than the applied force for injectable dosage forms (50 N), indicating the ease of administration by injection [33]. Therefore, based on the gel formation behavior and physicochemical properties studied, the most promising formulation to incorporate imatinib (IM) was at 30% *w*/*w* gamboge-based ISG.

### 2.2. Imatinib-Loaded 30% w/w Gamboge-Based ISGs

#### 2.2.1. Gel Formation

**Figure 3:**
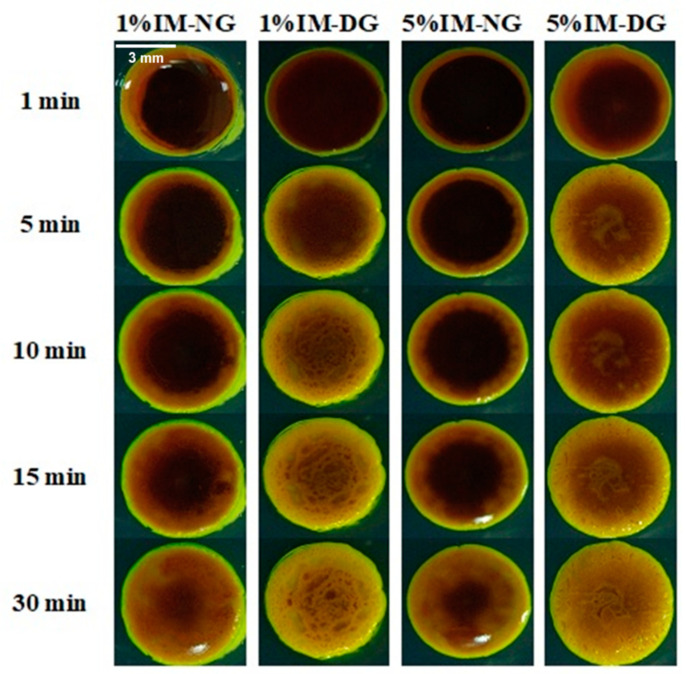
The matrix formation of IM-loaded 30% *w*/*w* gamboge ISGs in the agarose gel well under a stereomicroscope.

The gel formation of gamboge-based ISG formulations loaded with IM (imatinib) was observed under an inverted stereomicroscope, as shown in Figure 3. The formation of the opaque gel matrix started from the edge of the agarose gel and progressed toward the center over time. The rate of gel formation was found to depend on the type of solvent used and the IM content in the formulation. Specifically, ISGs formed faster in DMSO compared to NMP at the same concentration of IM, which was consistent with the drug-free ISG results. Moreover, higher IM content in the formulation resulted in a slower rate of matrix formation in both solvents. This was attributed to the high lipophilicity of IM (Log P 1.19), which enhanced the hydrophobicity of the system and consequently slowed down the matrix formation process [28,34].

#### 2.2.2. Interfacial Interaction

**Figure 4:**
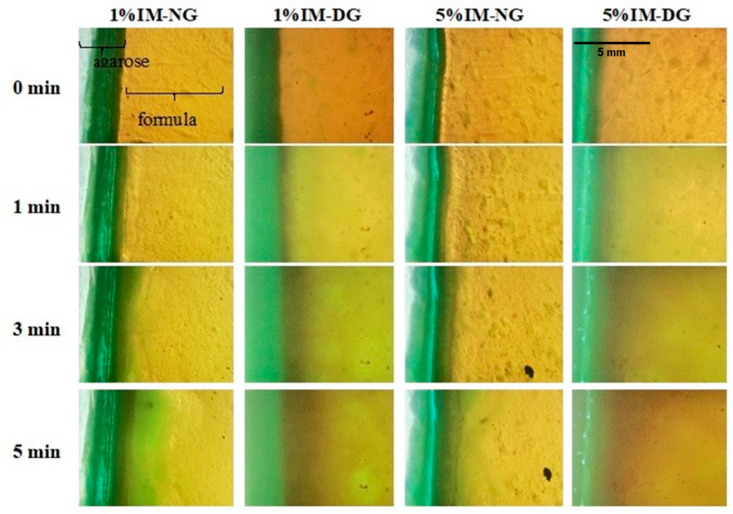
Interfacial phenomena of IM-loaded 30% *w*/*w* gamboge ISG systems after contact with aqueous phase from agarose gel under a fluorescence microscope at different time intervals.

The interfacial interaction between ISG solution and agarose gel was observed under an inverted microscope with the addition of sodium fluorescein for fluorescence tracking, resulting in a green color when viewed under a fluorescence microscope [35]. Upon contact of ISG solution with agarose gel, solvent exchange commenced, and the gel matrix started to form, which was observed as an increase in dark color over time due to the phase separation of gamboge into nontransparent matrix (Figure 4). This observation confirmed the previous results that matrix formation was faster in DMSO compared to NMP, and higher IM content in the formulation slowed down the matrix formation process. This obtained evidence can be attributed to the higher hydrophobicity of the system, as reported in previous studies [27,28].

#### 2.2.3. Rheological Property of IM-Loaded 30% *w*/*w* Gamboge ISG

**Figure 5:**
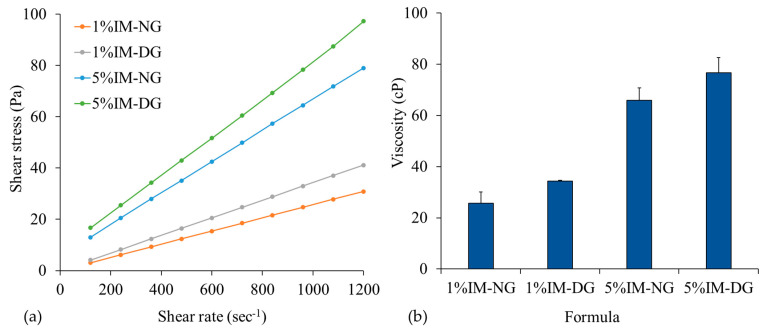
Plot of shear stress and shear rates (**a**) and viscosity (**b**) of IM-loaded 30% *w*/*w* gamboge-based ISG systems in NMP (N) and DMSO (D). (n = 3).

The rheological characterization of 1% and 5% *w*/*w* IM-loaded gamboge-based ISG formulations, including shear rate and viscosity, is shown in Figure 5a. It was observed that shear stress proportionally increased with an increase in shear rate, indicating Newtonian fluid behavior [36]. This implies that each ISG formulation has a constant viscosity and maintains a consistent flow behavior under normal conditions, which is consistent with previous findings on various ISG systems [35,37].

The viscosity of the ISG solution increased with higher IM content in the formulation, as shown in Figure 5b. The increased solute concentration in the solvent led to the formation of new bonds, reducing the ability of solvent molecules to freely move and resulting in higher viscosity values [38]. Interestingly, ISG formulations using DMSO as a solvent exhibited higher viscosity compared to those using NMP at the same concentration of IM. This finding is consistent with a previous study that reported lower viscosity with the use of bleached shellac in DMSO compared to NMP and 2-pyrrolidone [29]. Therefore, the higher lipophilicity of NMP favored the dissolution of gamboge, resulting in lower viscosity solutions [35]. Moreover, the solvent that shows a high affinity to the polymer can reduce the overall viscosity of the formulations [18].

#### 2.2.4. Injectability and Mechanical Property

**Figure 6:**
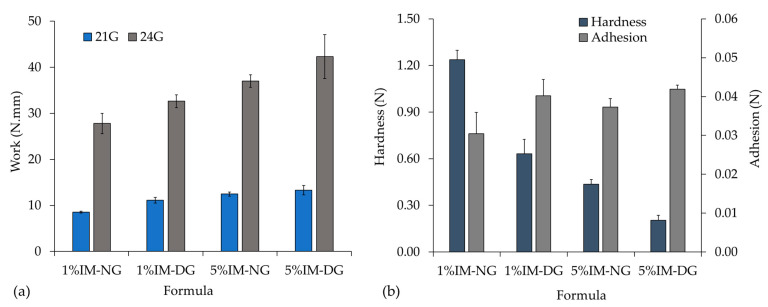
Injectability (**a**) and mechanical (**b**) property of IM-loaded 30% *w*/*w* gamboge-based ISG systems in DMSO (D) and NMP (N). (n = 3).

The injectability of ISG formulations was evaluated using two different sizes of gauge needles, No. 21 and No. 24. The required work for injecting the ISG solution through a No. 21 needle was lower compared to a No. 24 needle, due to the larger inner diameter of the needle, which reduces the resistance force [39]. Moreover, formulations with higher IM content required more work for injectability due to their higher viscosity, as shown in Figure 6a. It is important to note that the required work for injectability should be less than 50 N·mm for ease of injection [33]; all formulations in this study complied with this criterion, with applied forces lower than 50 N·mm. This is lower than the reported values for phase-inversion beta-cyclodextrin-based ISG systems [40].

The mechanical properties of aged ISG solutions in agarose wells after incubation for 3 days are presented in Figure 6b. The hardness of ISG decreased with increasing IM content in the formulation. ISG formulations prepared in NMP solvent exhibited higher hardness compared to those prepared in DMSO, which could be attributed to the slower matrix formation process of the system using NMP as a solvent, as previously mentioned [41], resulting in a dense and more compact ISG after 3 days of incubation. This finding is consistent with the gel formation study results that reported slower gel formation in NMP compared to DMSO. Moreover, there was no significant difference in adhesion force among all formulations, suggesting that neither the type of solvent nor the addition of IM had an impact on the adhesion force of the formulations.

#### 2.2.5. In Vitro Drug Release Study

**Figure 7:**
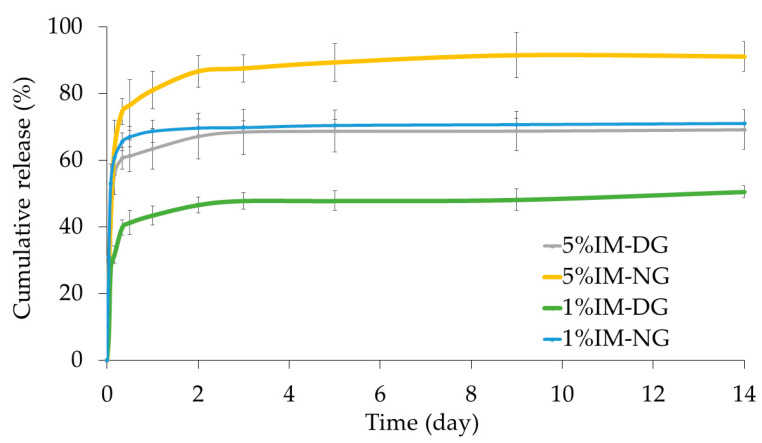
Release profile of IM from gamboge ISG systems (n = 3).

The release profile of IM from the 30% *w*/*w* gamboge-based ISG over time is shown in Figure 7. The release of IM was higher from ISG prepared in NMP compared to the DMSO formulation, and higher IM content showed a faster cumulative release compared to lower IM content. For instance, within 8 h, 40% of IM was released from the 1% *w*/*w* IM content, while 80% was released from the 5% *w*/*w* IM content. The order of cumulative drug release was found to be 1% IMDG < 5% IMDG < 1% IMNG < 5% IMNG, which is consistent with a previous study that reported faster release with higher content of paclitaxel in polymeric depot [42]. The initial burst release of IM was higher in NMP than in DMSO, possibly due to the higher affinity of DMSO with water. 

Upon contact with PBS, the ISG solution initiated the phase inversion process more rapidly, leading to a greater burst release of IM. However, later on, a denser depot structure was formed, making it difficult for water to influx and resulting in minimal drug release until gamboge matrix degradation occurred [43]. Overall, the cumulative release of IM prepared with NMP was higher due to the slower matrix formation process, resulting in a sponge-like surface with a porous cross-sectional structure [28]; nonetheless, these ISGs provided a sustained release of IM for up to 14 days. The release IM from our study showed longer sustained release and higher cumulative release than an IM-loaded liposomes system [44].

The kinetic release parameters, including the correlation coefficient (*r*^2^) and release exponent (*n*), were calculated, as shown in Table 2. The cumulative IM release profiles were fitted to various mathematical models, and the Korsmeyer-Peppas equation provided the highest *r^2^* values of 0.890–0.961 for the IM-loaded formulations, with *n* values of less than 0.45, indicating quasi-Fickian diffusion as the drug release mechanism [45,46]. Furthermore, consistent n values were observed within the same solvent, even with varying drug concentrations, suggesting that the type of solvent influenced the dissolution mechanism.

#### 2.2.6. Quantification of GA

The quantification of GA content in the gamboge resin was performed using high-performance liquid chromatography (HPLC) to elucidate the cytotoxicity of the formulations based on GA content, following our previously validated method [47]. External standard solutions of GA in the concentration range of 5–120 μg/mL were used to generate a standard curve. The GA content in the samples was calculated using the standard curve equation: y = 11.803x + 3.1421 (*r*^2^ = 0.9998). The GA content in the gamboge resin determined in this study was 44.42 ± 0.125 mg/100 mg of gamboge, which was significantly higher than the previous study, which reported 25.75 ± 4.89 mg of GA/100 mg in natural gamboge resin [48].

#### 2.2.7. GA Release

**Figure 8:**
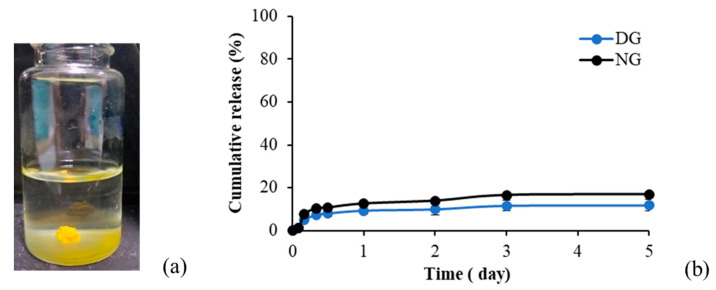
Gel formation of gamboge ISG system in the release medium after 1 h (**a**) and release profile of GA from gamboge ISG systems (**b**). (n = 3).

The gel formation of 30% *w*/*w* gamboge-based ISG in the release medium, and the cumulative release of GA from 30% *w*/*w* gamboge ISG using DMSO and NMP as solvents, are shown in Figure 8a and 8b, respectively. The release of GA was gradual during the initial day and remained sustained throughout the 5-day investigation period. The cumulative release of GA from the ISG solution in NMP was slightly higher than that in DMSO (16% and 11%, respectively). The low release of GA from ISG was attributed to the very poor water solubility of GA in aqueous solutions (<5 ppm), which suggests the addition of tween 80 to improve the solubility of GA for drug release studies [49]. Another study also reported incorporating GA into micelles, resulting in a significant increase in GA solubility and achieving 90% release after 4 days [50].

#### 2.2.8. SEM Morphology Study

**Figure 9:**
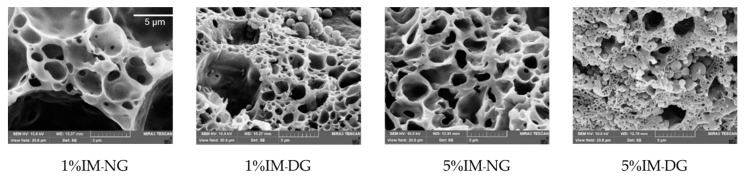
Cross-sectional SEM images of IM-loaded 30% *w*/*w* gamboge-based ISG systems in DMSO (D) and NMP (N). (10,000×).

The cross-sectional morphology of the 30% *w*/*w* gamboge-based ISG systems was examined using SEM analysis (Figure 9). It was observed that all formulations exhibited a spherical morphology with particle sizes of less than 5 μm on the surface view, which might be the IM agglomeration during the phase transformation process of ISG. The interconnected porous structure was particularly evident in DMSO-based gamboge ISG formulations containing 1% and 5% IM. The presence of solvent-water exchange resulted in the formation of an asymmetric structure with a dense and compact skin layer accompanied by channel- and finger-like structures, as reported by Zare (2008) [28]. A more porous structure was observed in the 1% and 5% IMNG formulations. This finding is consistent with the study by Liu et al. (2012) [43], who reported that ISG formulations with pure DMSO exhibited a denser structure compared to lower hydrophilicity systems such as NMP, resulting in more swelling and a porous structure that facilitated the leaching out of the entrapped compound. This elucidates the reason why the cumulative drug release in Section 2.2.5 was greater in the gamboge-based ISG prepared using NMP as the solvent.

#### 2.2.9. Cytotoxicity Activity

##### Gamboge Resin Cytotoxicity

**Figure 10:**
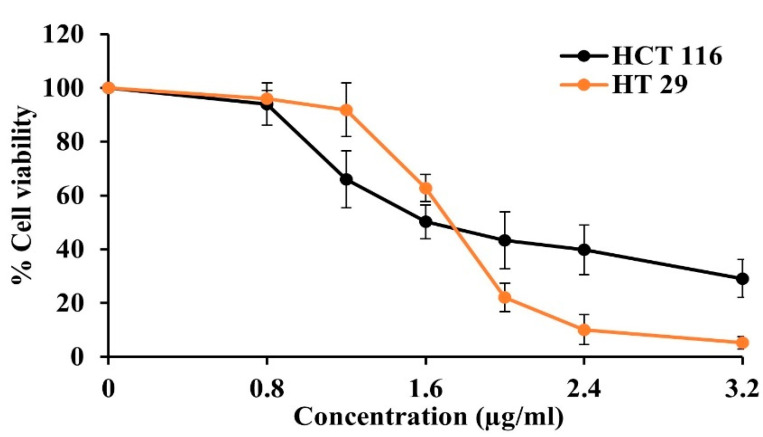
% cell viability of gamboge resin against HCT116 and HT29 cell lines.

The cytotoxicity of gamboge resin against HCT116 and HT29 cells was tested using the WST-1 assay. After 72 h of treatment with gamboge samples, the percentage of cell viability was determined and is shown in Figure 10. The cell viability decreased with increasing concentration of gamboge, indicating a dose-dependent cytotoxic activity of the samples. The IC_50_ values of gamboge against HCT116 and HT29 cells were 2.7 µM and 2.95 µM, respectively. These values were higher than previous reports on the cytotoxicity of gamboge resin against the HCT116 cell line (0.5–0.64 µM) [51,52]. This difference may be attributed to variations in the sources of gamboge resin and the content of GA, which was not quantified in those papers. However, the cytotoxicity of gamboge resin was still higher than that of IM, which has been reported to have IC_50_ values of 7.5 µM and 21.98 µM against HCT116 and HT29 cell lines, respectively [53]. In addition, it is also more potent than other natural products, such as propolis and benzoin, by showing significantly lower IC_50_ values [41]. Therefore, due to the interesting cytotoxicity of gamboge, it is worthwhile to consider using gamboge as an in situ gel (ISG) in this study.

##### IM-Loaded Gamboge-Based ISG Cytotoxicity

**Figure 11:**
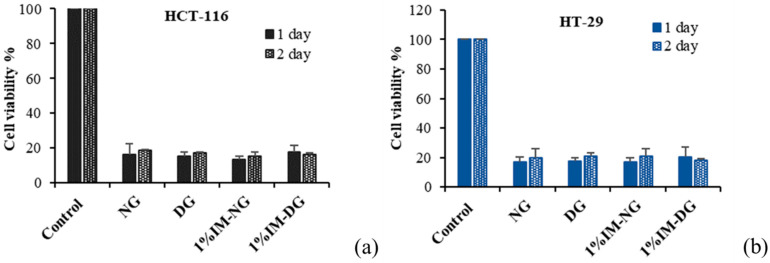
Cell viability of HCT-116 (**a**) and HT-29 (**b**) cells treated with extracted medium from IM-loaded gamboge-based ISG formulations after incubation at 1 and 2 days.

The cytotoxicity of the ISG formulations containing 1% IM in DMSO and NMP against HCT116 and HT29 cell lines was investigated, and the results are shown in Figure 11. Formulations without IM were also tested to investigate potential synergistic effects. The DMEM medium extracted from drug-free and 1% IM-loaded formulations after 1 and 2 days of incubation was administered to HCT116 and HT29 cells. The percentage of cell viability was subsequently analyzed after 72 h of incubation. The results revealed that the percentage of cell viability was significantly decreased in both IM-containing and non-IM-containing formulations compared to the negative control (*p* < 0.05). This could be attributed to the well-documented cytotoxic properties of the gamboge resin, as described in the Section “Gamboge Resin Cytotoxicity”. However, there was no significant difference in the percentage of cell viability among the ISG samples (*p* > 0.05). Therefore, the cytotoxicity of the developed ISG formulations may be mainly contributed by gamboge. Further investigation could be conducted to confirm the potential synergistic effect between gamboge and IM.

**Figure 12:**
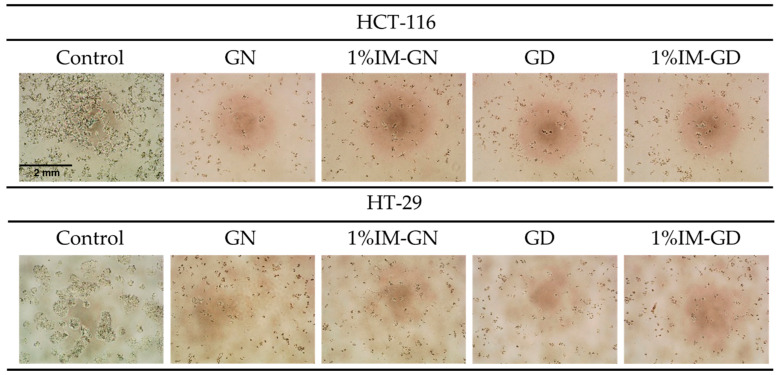
Morphology of HCT-116 and HT-29 cells treated with control and extracted medium from gamboge-based ISG formulations after incubation 1 day using an inverted microscope (40×) measured with WST-1 assay.

The morphology of HCT116 and HT29 cells was observed under an inverted microscope after incubation with the extracted medium from ISG formulations at day 1 using the WST-1 assay. The results, as shown in Figure 12, were consistent with the cytotoxicity test results, indicating that cells treated with all ISG formulations exhibited cell shrinkage, indicating non-viable cells, which was significantly different from the negative control (DMEM) where cells exhibited normal growth. Nevertheless, it was challenging to distinguish viable cells among those treated with ISG formulations. Based on all the results, it can be concluded that natural gamboge resin and the developed ISG formulations possess cytotoxicity against the tested colon cancer cell lines HCT116 and HT29.

## 3. Conclusions

The developed in situ gel (ISG) based on gamboge resin in DMSO and NMP solvents showed promising results. The formulation with 30% *w*/*w* gamboge-based ISG exhibited favorable gel formation behavior and physicochemical properties, making it suitable for incorporating IM. The IM-loaded 30% *w*/*w* gamboge-based ISG demonstrated sustained release of IM for up to 14 days, with a release mechanism profile consistent with quasi-Fickian diffusion. Furthermore, the gamboge-based ISG prepared in NMP displayed a highly porous morphological structure, as observed through SEM, leading to a greater cumulative and sustained release of IM compared to formulations prepared in DMSO. Moreover, natural gamboge resin exhibited potent cytotoxicity against the colorectal cancer cell lines HCT116 and HCT29. Although the cytotoxicity of the developed ISG formulations, both loaded and unloaded with IM, were not significantly different, this confirms the potent cytotoxic effect of all the developed gamboge-based ISG formulations. Considering the rheological and mechanical properties of these formulations, the gamboge ISG system holds the potential to be an injectable gel that can effectively extend the release of IM.

## 4. Materials and Methods

### 4.1. Materials

The gamboge resin was obtained from a nearby drug store in Thailand, while HPLC grade acetonitrile, methanol, and orthophosphoric acid (Lab Scan) were acquired from CT Chemical Ltd. in Bangkok, Thailand. The imatinib mesylate and gambogic acid used in the study were obtained from SWY Biotech Co. in Dongguan, China, and Chengdu Biopurify Phytochemicals Ltd. in Chengdu, China, respectively.

### 4.2. Preparation of Gamboge ISG

First, the 25, 30, 40, and 50% *w*/*w* gamboge were dissolved in DMSO and NMP. IM at 1% and 5% *w*/*w* was dissolved in NMP or DMSO using a stirrer. Next, 30% *w*/*w* gamboge was added to the solution and stirred until it was completely dissolved. All formulations were assigned as shown in Table 3.

### 4.3. Physicochemical Study

#### 4.3.1. Rheological Behavior

Gamboge ISGs were investigated for their rheological behaviors at 25 °C with a rheometer (Physica MCR; Anton Paar, Ashland, VA, USA) using a 25-mm diameter plate size, measured at various shear rates.

#### 4.3.2. Injectability

The injectability of the ISG sample was measured using a texture analyzer (TA.XT plus, Stable Micro Systems, Godalming, UK) in compression mode. The sample was placed in a 1 mL plastic syringe connected to a 24- and 27-gauge needle, which was clamped to a stainless-steel stand. The upper probe of the texture analyzer was moved downward at a constant speed of 1.0 mm/s and a constant force of 0.1 N until a displacement of 20 mm was reached, and the injectability force was recorded. The area under the curve was calculated as the work force. The measurements were performed in triplicate.

#### 4.3.3. Mechanical Property

A sample of 150 µL was placed in an agarose well and left for 24 h to ensure complete matrix formation from solvent exchange between DMSO or NMP from formulation and PBS pH 7.4 from agarose gel. An analytical probe, affixed to a texture analyzer, was lowered onto the matrix at a rate of 0.5 mm/s and held in contact with the matrix for 60 s. The probe was then lifted at a speed of 10 mm/s. The maximum force at which the probe penetrated the sample was recorded as the maximum deformation force (i.e., the hardness), while the upward movement of the probe between the surface of the sample and the probe was recorded as the adhesion force. The measurements were performed in triplicate.

### 4.4. Gel Formation

The transformation of ISG solutions into a semisolid-like layer was visually observed after injecting them into the test tubes containing PBS (pH 7.4). Photographs were captured at various time intervals starting from the initial transformation step until the formation of the matrix was fully accomplished. In addition, the gel formation characteristics were studied using microscopic observation. A 6-mm agarose (0.6% *w*/*w*) well was filled with 150 µL of ISGs, and the matrix transformation behavior was recorded at different time intervals using a stereomicroscope (SZX2-ILLD, OLYMPUS corp., Tokyo, Japan).

### 4.5. In Vitro Drug Release

A total of 0.05 g of ISGs were injected into 10 mL of PBS (pH 7.4) at 37 °C with an agitation speed of 50 rpm. At different time points, 2 mL of the dissolution medium was withdrawn and replaced with the same amount of fresh PBS (pH 7.4). The released IM was quantified using Agilent 1220 Infinity HPLC (C8 column, Dr. Maisch, 4.6 × 150 mm, 5 µm) at a flow rate of 1 mL/min with a mixture of acetonitrile and 0.1% orthophosphoric acid (17:83). The experiment was performed in triplicate. To study the release mechanisms, the release profile was fitted to various release mechanism models (zero-order, first-order, Higuchi square root of time, and power law) using DD-solver software.

### 4.6. SEM Image Analysis

The freeze-dried ISG samples after 14 days released in PBS (pH 7.4) were coated with gold and examined using a scanning electron microscope (Maxim 200 Camscan, Cambridge, UK) with an accelerating voltage of 15 kV.

### 4.7. Cytotoxicity Test of Gamboge and ISGs

The cytotoxicity of the gamboge and 1% *w*/*w* IM-loaded ISG system was assessed against HCT 116 and HT29 cell lines using the medium extraction method. ISG samples were formed in DMEM at 37 °C, and the media was collected on day 1 and day 5 of incubation. Then, 5000 cells of HCT116 and HT29 were seeded into a 96-well plate and incubated in DMEM for 1 day. The media was then replaced with 100 µL of the extracted media, and after 72 h, the survival cells were assessed using the WST-1 assay. The absorbance was measured at 430 nm using a Nivo^®^ microplate reader (Perkin, London, UK). All tests were performed in quadruplicate (n = 4).

### 4.8. Statistical Analysis

The collected data were analyzed for statistical significance using SPSS software version 11.5, and reported as mean and standard deviation (S.D.). Differences among groups were tested by one-way ANOVA, followed by post hoc analysis using the LSD method at a significance level of 95% (*p* < 0.05).

## Figures and Tables

**Table 1:** Physicochemical properties of 25 to 50% *w*/*w* gamboge-based ISGs containing in NMP and DMSO. (n = 3).

Formula	pH	Viscosity (cP)	Injectability	
			Force (N)	Work (N·mm)
NG25	7.86 ± 0.06	38.10 ± 0.00	0.78 ± 0.11	2.60 ± 0.17
NG30	7.79 ± 0.05	43.66 ± 0.00	0.90 ± 0.04	3.21 ± 0.61
NG40	7.58 ± 0.06	62.97 ± 0.46	1.07 ± 0.14	3.13 ± 1.11
NG50	7.34 ± 0.05	ND	1.07 ± 0.28	5.66 ± 0.49
DG25	6.86 ± 0.12	23.81 ± 0.00	0.68 ± 0.15	1.53 ± 0.25
DG30	6.84 ± 0.03	39.69 ± 0.00	0.86 ± 0.13	2.85 ± 0.36
DG40	6.80 ± 0.02	132.60 ± 0.00	1.10 ± 0.08	5.73 ± 0.51
DG50	6.25 ± 0.21	810.03 ± 3.33	1.38 ± 0.07	4.55 ± 0.53

ND = Not determined.

**Table 2:** Kinetics modeling release profile of IM from gamboge-based ISG systems in DMSO and NMP solvents.

Formula	Zero Order	First Order	Higuchi Order	Korsmeyer-Peppas		
	*r* ^2^	*r* ^2^	*r* ^2^	*r* ^2^	*n*	Release Mechanism
1% IM-NG	0.269	0.600	0.756	0.951	0.234	quasi-Fickian diffusion
1% IM-DG	0.509	0.672	0.889	0.906	0.402	quasi-Fickian diffusion
5% IM-NG	0.341	0.721	0.862	0.890	0.246	quasi-Fickian diffusion
5% IM-DG	0.454	0.903	0.924	0.961	0.366	quasi-Fickian diffusion

**Table 3:** The composition of IM-loaded gamboge ISG formulations.

Formula	Conc. (% *w*/*w*)			
	IM	Gamboge	NMP	DMSO
NG25		25	75	
NG30		30	70	
NG40		40	60	
NG50		50	50	
DG25		25		75
DG30		30		70
DG40		40		60
DG50		50		50
1%IM-GN	1	30	69	-
1%IM-GD	1	30	-	69
5%IM-GN	5	30	65	-
5%IM-GD	5	30	-	65

## Data Availability

The data presented in this study are available on request from the corresponding author.
